# The Antioxidant and Proapoptotic Effects of *Sternbergia clusiana* Bulb Ethanolic Extract on Triple-Negative and Estrogen-Dependent Breast Cancer Cells In Vitro

**DOI:** 10.3390/plants12030529

**Published:** 2023-01-24

**Authors:** Mona El Samarji, Maria Younes, Marianne El Khoury, Tony Haykal, Nazira Elias, Natalia Gasilova, Laure Menin, Ahmad Houri, Nisrine Machaka-Houri, Sandra Rizk

**Affiliations:** 1 Department of Natural Sciences, Lebanese American University, Byblos P.O. Box 36, Lebanon; 2 Institute of Chemical Sciences and Engineering, Ecole Polytechnique Fédérale de Lausanne (EPFL), 1015 Lausanne, Switzerland; 3 Department of Natural Sciences, Lebanese American University, Beirut 1102-2801, Lebanon; 4 Department of Life and Earth Science, Faculty of Sciences, Saint Joseph University, Ras Maska 1104-2020, Lebanon

**Keywords:** breast cancer, *Sternbergia clusiana*, phytochemicals, apoptosis, antioxidant

## Abstract

Background: *Sternbergia clusiana* belongs to the Amaryllidaceae family and is recognized for the valuable biological activity of its major bioactive compounds. The aim of the current is to evaluate the anticancer effects of the ethanolic bulb extract of *Sternbergia clusiana* (ScBEE) on breast cancer cells in vitro and to further reveal the underlying cellular mechanism. Methods: An MTS cell viability assay was performed on MDA-MB-231 and MCF-7 cells, along with cell cycle analysis, cell death ELISA, Western blot analysis and an ROS production assay to decipher the mechanism of death. LC-MS/MS was also performed to identify the chemical composition of this ethanolic extract. Results: The results show a selective antiproliferative effect on both cell lines with no effect on normal mesenchymal stem cells. Further analysis suggested the activation of the apoptotic pathway as reflected by the increase in cellular and DNA fragmentation and alterations in apoptotic proteins such as Bax, Bcl-2 and c-PARP. ScBEE was also found to exhibit antioxidant effect, as shown by a decrease in ROS production. The underlying mechanism of action was explained by the presence of several bioactive compounds identified by LC-MS/MS, including alkaloids, terpenoids and phenols, which are elaborated in the manuscript. Conclusion: This study highlights the antioxidant and anticancerous properties of *S.clusiana* for breast cancer treatment.

## 1. Introduction

Breast cancer (BC) was reported to be the most common type of cancer diagnosed in women and the leading cause of women’s death worldwide based on epidemiological studies conducted by the International Agency for Research on Cancer (IARC) [1]. Invasive ductal and lobular carcinoma are the two major types that are broadly used to describe BC. These cells can become invasive and spread to other tissues of the body when subjected to further events of multiplication, propagation and infiltration [2]. Molecular classification of BC depends on the presence of hormone receptors, i.e., estrogen receptor (ER), progesterone receptor (PR) and human epidermal growth factor receptor-2 (HER2). Two different BC cell lines, MDA-MB-231 and MCF7, were established from patients with invasive ductal carcinoma. Hormone-independent MDA-MB-231 cells are identified as triple-negative breast cancer (TNBC) cells because they lack ER, PR and HER2 expression. However, MCF7 cells are hormone-dependent mammary cells that could be directly stimulated by estrogen [3].

Despite the wide range of modern medical practices employed nowadays to treat severe cases of BC tumors characterized by a heterogenous expression of cellular biomarkers [4], the use of complementary and alternative medicine (CAM) is becoming increasingly frequent [5]. One common modality of CAM is ayurvedic therapy, an ancient Indian practice based on the use of natural compounds and medicinal plants to suppress tumorigenesis [6]. Considering the unspecific targeting mode, the prohibitive cost and the countless short- and long-term side effects of conventional cancer therapies [7], secondary metabolites derived from the plant kingdom have been thoroughly investigated for their anticancerous properties. These phytochemical compounds were assessed for their safety by evaluating their cytotoxic effect on normal cells. Various plant extracts and natural compounds were previously found to exhibit a selective effect on cancer cells without affecting normal cells extracted from rats or human bone marrow [8,9].

*Sternbergia clusiana* (Ker Gawl.) Ker Gawl ex Spreng is a bulbous perennial flowering species characterized by a yellow wine-glass shape and a well-developed perigonium [10]. The genus Sternbergia belongs to a distinctive family of medicinal plants, *Amaryllidaceae*, possessing a large variety of potent pharmacological isoquinoline alkaloids, of which lycorine is a major compound [11]. These structurally unique alkaloids found almost exclusively in Amaryllidaceae were established to have cytotoxic and chemotherapeutic properties, in addition to their interference with biological and synthetic pathways of many crucial proteins involved in multiple diseases [12,13]. 

Showing a considerable total amount of phenols and flavonoids, most *Sternbergia* members were identified as antioxidant species with free radical scavenging activity [14]. The antioxidant effect of *Sternbergia* was previously revealed in several subspecies of *S. lutea* that also showed an in vitro cytotoxic activity and anti-inflammatory properties, in addition to an antidiabetic effect detected through the inhibition of α-glucosidase [15,16]. Additional studies reported the presence of alkaloids in Sternbergia species known for their potent role in treating diseases. In a study conducted by Tanker et al., *S. clusiana* was found to exhibit analgesic activity due to the presence of the major alkaloids, lycorine and hemanthamine [17]. Other studies have reported that *S. clusiana* species are important sources of the anticholinesterase agent galantamine, currently known for being highly effective in treating Alzheimer disease [18,19]. Besides its antifungal properties [20], *S. clusiana* was also suspected to have anticancerous potential due to its richness in *Amaryllidaceae* alkaloids (AA) such as lycorine [17,21], which was previously shown to be effective in the regulation of malignant cell invasion and metastasis [22]. Another study conducted by Sun et al. reported its role in inducing apoptosis in non-small cell lung carcinoma (NSCLC) by interfering with Wnt/β-catenin signaling and reversing epithelial–mesenchymal transition (EMT) [23]. A potential approach to halt the spread of cancer is the use of natural chemicals in chemoprevention. However, no studies have been performed to investigate the cytotoxic effect and to decipher the mechanism of action of *S. clusiana* on different cancer cell lines.

In the current study, we aimed to investigate the anticancer properties of *Sternbergia clusiana* bulb ethanolic extract (ScBEE) on BC cells in vitro and to reveal the mechanism of action.

## 2. Results

### 2.1. ScBEE Exerts a Selective Antiproliferative Effect on MDA-MB-231 and MCF-7 Cells Compared to Its Effect on MSCs

**Figure 1:**
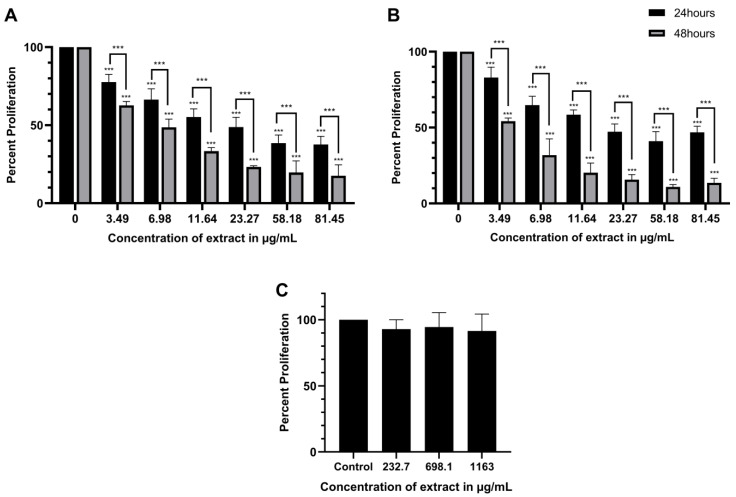
Antiproliferative effect of ScBEE on breast cancer cell lines and normal mesenchymal stem cells. A significant dose- and time-dependent decrease in the proliferation of (**A**) MDA-MB-231 and (**B**) MCF-7 cell lines was observed, with no cytotoxic effect on (**C**) normal mesenchymal stem cells (MSCs) when treated with high doses of ScBEE. Significant differences are reported; *** indicates a *p*-value of 0.0001 < *p* < 0.001.

To elucidate the cytotoxic effect exerted by ScBEE, MDA-MB-23, MCF-7 and MSCs were treated with increasing concentrations of ScBEE for 24 and 48 h, followed by the addition of an MTS proliferation reagent. Viability decreased significantly in MDA-MB-231 in a dose- and time-dependent manner, reaching 37.6% (*p*-value < 0.0001) and 17.6% (*p*-value < 0.0001) proliferation when treated with the highest concentration of ScBEE (81.445 μg/mL) for 24 h and 48 h, respectively (Figure 1A). Similarly, a significant effect was observed on MCF-7, whereby cell proliferation decreased significantly when treated with 81.445 μg/mL ScBEE, reaching 46.9% (*p*-value < 0.001) and 13.6% (*p*-value < 0.0001) at 24 h and 48 h, respectively (Figure 1B). The half-maximal inhibitory concentration (IC_50_) was calculated using GraphPad Prism 8 software, and the IC_50_ of MDA-MB-231 was found to be 21.55 μg/mL (0.926% *v*/*v*) at 24 h and 6.72 μg/mL (0.289% *v*/*v*) at 48 h, whereas the IC_50_ of MCF-7 cells was found to be 23.08 μg/mL (0.992% *v*/*v*) at 24 h and 3.72 μg/mL (0.160% *v*/*v*) at 48 h (Table 1). MSCs are currently used as an appropriate model of normal cells to check the selectivity of natural products [24,25,26]. Interestingly, ScBEE showed no cytotoxic effect on normal MSCs when treated with highly saturated doses of the extract, highlighting its selective effect on malignant cells (Figure 1C). The remaining experiments were performed on MDA-MB-231 and MCF-7 cells treated with ScBEE for 24 h using the closest concentrations to the IC_50_ (0.15, 0.5 and 2.5% *v*/*v*). 

### 2.2. ScBEE Induces Cellular Fragmentation of BC Cells

**Figure 2:**
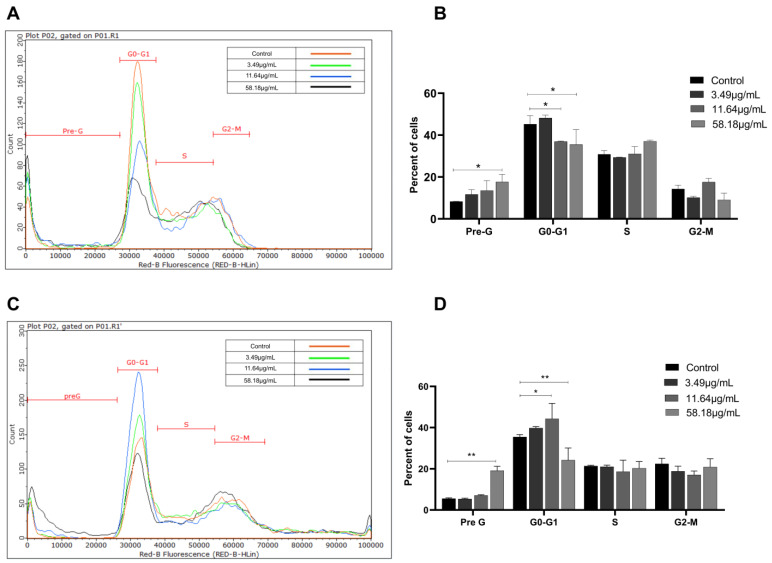
ScBEE induces cellular fragmentation in a dose-dependent manner in breast cancer cell lines. Cell cycle analysis of (**A**) MDA-MB-231 and (**C**) MCF-7 cells treated with increasing concentrations of ScBEE for 24 h. A significant dose-dependent decrease in G0-G1 phase along with a significant increase in the pre-G phase in both cell lines, (**B**) MDA-MB-231 and (**D**) MCF-7 was noticed after 24 h of treatment. Significant differences are reported; * indicates a *p*-value of 0.01< *p* < 0.05, ** indicates a *p*-value of 0.001 < *p* < 0.01.

To determine the effect of ScBEE on cell cycle progression, MDA-MB-231 and MCF-7 cells were stained with propidium iodide (PI) followed by flow cytometric analysis after treatment with increasing concentrations of the extract for 24 h. Cells were distributed into four phases (pre-G0, G0-G1, S and G2-M) based on their DNA content. The results showed a significant increase in the percentage of cells present in the pre-G0 phase (DNA < 2 *n*) from 8.3% to 17.6% and from 5.6% to 19.1% when MDA and MCF cells were treated with the highest concentration of ScBEE (58.18 μg/mL), respectively. Additionally, a decrease in the G0-G1 phase (2 *n* < DNA < 4 *n*) from 45.3% in control samples to 35.6% was noticed upon 58.18 μg/mL ScBEE treatment of MDA cells (Figure 2A,B). Similarly, MCF-7 cells showed a significant decrease in the percentage of cells in the G0-G1 phase from 35.5% to 24.2% upon treatment with the highest dose (Figure 2C,D). These results suggest that ScBEE promotes cellular fragmentation of MDA-MB-231 and MCF-7 cells upon treatment with ScBEE for 24 h. 

### 2.3. ScBEE Promotes DNA Fragmentation in Breast Cancer Cells

**Figure 3:**
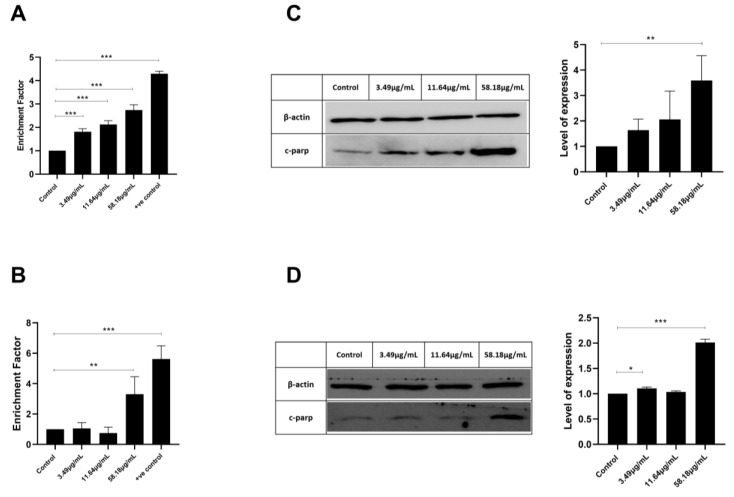
ScBEE induces DNA fragmentation in a dose-dependent manner as quantified via cell death detection ELISA in (**A**) MDA-MB-231 and (**B**) MCF-7 cells. Western blot analysis revealed the increased expression of cleaved PARP with increased treatment concentration for 24 h in (**C**) MDA-MB-231 and (**D**) MCF-7 cells. Significant differences are reported; * indicates a *p*-value of 0.01 < *p* < 0.05, ** indicates a *p*-value of 0.001 < *p* < 0.01 and *** indicates a *p*-value of 0.0001 < *p* < 0.001. The full-length blots of all trials are reported as Supplementary Figures S1 and S2.

In order to determine the mechanism by which ScBEE exerts its cytotoxic effect on both BC cell lines, cell death ELISA was performed. This assay allows for the quantification of DNA fragmentation, a major hallmark of apoptosis. Quantification was performed by calculating the enrichment factor, which is the ratio of the absorbance of treated cells to that of untreated cells. As illustrated in Figure 3, a significant increase in DNA fragmentation was noticed in both BC cell lines. MDA-MB-231 cells showed a 2.7-fold increase at the highest concentration of ScBEE used. Similarly, MCF-7 showed a significant 3.3-fold increase when treated with 58.18 μg/mL extract for 24 h. These findings were compared to a positive control inducing DNA fragmentation represented by a 4.29-fold increase and 5.6-fold increase as compared to untreated MDA-MB-231 and MCF-7 cells, respectively.

In order to understand what might be responsible for the DNA fragmentation, Western blot analysis was performed to quantify the expression of the cleaved form of PARP highly involved in DNA repair. The results revealed an upregulation in cleaved PARP (c-PARP) expression in both cell lines, MDA-MB-231 and MCF-7 cells (Figure 3C,D).

### 2.4. ScBEE Activates the Apoptotic Pathway via the Activation of the Mitochondrial Pathway

**Figure 4:**
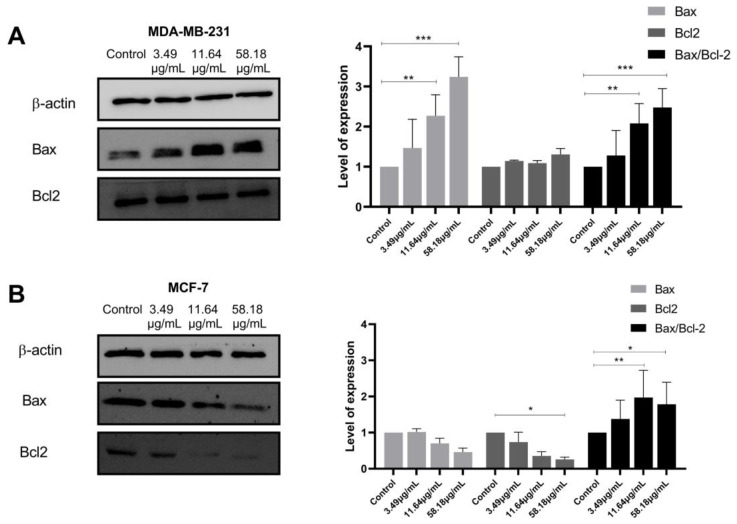
ScBEE activates the mitochondrial apoptotic pathway. Western blot analysis shows the upregulation of the Bax–Bcl2 ratio upon treatment of (**A**) MDA-MB-231 and (**B**) MCF-7 cells with increasing concentrations of the extract. Significant differences are reported; * indicates a *p*-value of 0.01 < *p* < 0.05, ** indicates a *p*-value of 0.001 < *p* < 0.01 and *** indicates a *p*-value of 0.0001 < *p* < 0.001. The full-length blots of all trials are reported as Supplementary Figures S3–S6.

**Figure 5:**
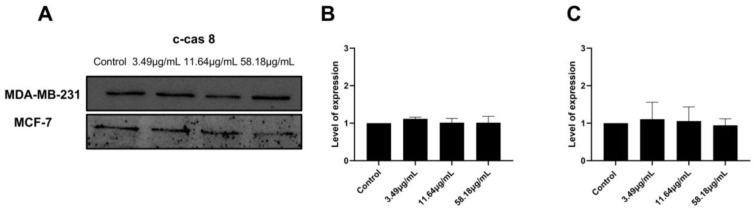
ScBEE does not activate the extrinsic pathway. Western blot analysis (**A**) showed no significant effect of the extract on the expression of c-caspase 8 in (**B**) MDA-MB-231 and (**C**) MCF-7 cells. Significant differences are reported. The full-length blots of all trials are reported as Supplementary Figures S7 and S8.

To investigate the proteins involved in the apoptotic signaling pathway triggered by ScBEE in MDA-MB-231 and MCF-7 cell lines, Western blot analysis was carried out. The expression of the mitochondrial-membrane-regulating proteins, Bax and Bcl2, was quantified, revealing a significant increase in the Bax/Bcl2 ratio in both cell lines when the cells were treated with increasing concentrations of the extract for 24 h (Figure 4A,B). Furthermore, the expression of the initiator caspase was measured in MDA-MB-231 and MCF-7 cells. No significant effect was exerted on the expression of cleaved caspase 8 in both breast cancer cell lines (Figure 5). These results suggest the activation of the intrinsic mitochondrial pathway in both cell lines, MDA-MB-231 and MCF-7 cells, in response to the ScBEE. 

### 2.5. ScBEE Inhibits ROS Levels in Breast Cancer Cells

**Figure 6:**
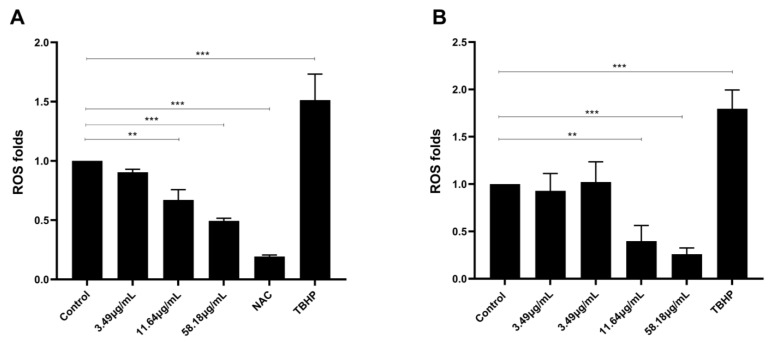
Antioxidant properties of ScBEE in breast cancer cell lines. Measurement of reactive oxygen species production in (**A**) MDA-MB-231 and (**B**) MCF-7 cells showed a significant decrease, resulting in increased concentrations of ScBEE. TBHP and NAC were used as positive and negative controls, respectively. Significant differences are reported; ** indicates a *p*-value of 0.001 < *p* < 0.01 and *** indicates a *p*-value of 0.0001< *p* < 0.001.

To study the effect of ScBEE on oxidative stress, the levels of reactive oxygen species were measured upon treating MDA-MB-231 and MCF-7 cells with increasing concentrations of Sternbergia extract, a potent ROS inducer (TBHP) or an effective ROS inhibitor (NAC), for 24 h. Results showed a significant dose-dependent decrease to 0.49-fold when MDA-MB-231 cells were treated with 58.18 μg/mL ScBEE, indicating a downregulation in ROS production. Similarly, MCF-7 cells showed a significant decrease, reaching 0.4-fold upon treatment with 58.18 μg/mL extract (Figure 6). These values were compared to the levels of ROS reached upon treating both cell lines with the negative control (NAC) and the positive control (TBHP). This downregulation of ROS reveals the antioxidant potential of ScBEE in breast cancer cell lines.

### 2.6. Chemical Characterization of Sternbergia Bulb Ethanolic Extract Using LC-MS/MS

Liquid chromatography coupled with tandem mass spectrometry (LC-MS/MS) was performed in order to explore the chemical composition of the ethanolic extract of S. clusiana. A variety of bioactive compounds, such as benzofurans, linoleic acid derivatives, quinoline derivatives, phenols and flavonoids, was detected (Table 2). Several alkaloid derivatives were targeted for identification in ScBEE (Supplementary Table S1), and lycorine (RT = 2.4 min), a known Amaryllidaceae alkaloid, was found. Similarly, flavonol derivatives, namely quercetin (RT = 16.99 min) and rhamnetin (RT = 18.91 min), as well as linoleic acid derivatives, such as jasmonic acid (RT = 24.84 min) and corchorifatty acid F (RT = 36.33 min), were observed in this ethanolic bulb extract. Other major compounds detected by LC-MS/MS include trans-anethole (RT = 34.62 min) and ferulic acid (RT = 11.45 min).

## 3. Discussion

Over the past few decades, phytochemical research has been tremendously expanded, aiming to identify innovative natural components to be implemented in the treatment of complicated multifactorial diseases including cancer [27]. In addition to their anti-tumoral protective effect, several medicinal foods and plants have been shown not only to inhibit the proliferation of malignant cells, metastasis and angiogenesis but also to promote programmed cell death mechanisms, mainly apoptosis [28]. Commonly known for their structurally unique constituents, Amaryllidaceae species were extensively inspected in order to investigate the prominent medicinal value of the abundant phytochemical compounds they contain [29]. Among the numerous plant genera that belong to the Amaryllidaceae species, members of the genus Sternbergia have always been considered prolific reservoirs for powerful bioactive compounds [16,30]. The aim of the current study was to examine *Sternbergia clusiana* bulb ethanolic extract (ScBEE) for its antiproliferative effect on breast cancer cells, MDA-MB-231 and MCF-7, and to determine its mode of action at the molecular level.

Based on previous studies, aqueous and alcoholic extracts from bulbs, fruits, stems and leaves of Amaryllidaceae plants were shown to display a significant anticancer effect by inhibiting the proliferation of a wide range of carcinoma (HeLa, MCF-7, A549 and LNCaP) and adenocarcinoma (MDA-MB-231 and PC3) cell lines [31,32]. These findings support the results we obtained in the cell viability assay that suggested a time- and dose-dependent cytotoxic effect of ScBEE on the two BC cell lines, MDA-MB-231 and MCF-7, with similar IC_50_ concentrations after 24 h but slightly smaller effect on MCF-7 than on MDA-MB-231 after 48 h of treatment. The extract selectivity in targeting malignant cells was also demonstrated by a non-significant antiproliferative effect on MSCs, indicating a potent resistance against highly concentrated doses of ScBEE, supporting the advantage of plant-based therapeutic agents over non-selective chemically derived competitors.

In order to identify the characteristics of the early underlying molecular mechanism that led to the prominent death detected at 48 h, the subsequent experiments were carried out on each of the BC cell lines after 24 h of incubation with ScBEE.

Latest findings have revealed that apoptosis may be a major pathway leading to cancer cell death initiated by Amaryllidaceae alkaloids [33,34]. Interestingly, the induction of apoptosis in ScBEE-treated breast cancer cells was clearly established by several hallmarks detected at the molecular and cellular levels. Cancer cell fragmentation was confirmed through cell cycle analysis; a significant increase in MDA-MB-231 and MCF-7 cells was identified during the pre-G phase (DNA < 2 n) upon treatment for 24 h with ScBEE. Another apoptosis-specific feature that was also shown to be induced by ScBEE on BC in our experiments is the internucleosomal cleavage of DNA into conserved size fragments [35] detected by cell death ELISA. A main provoker of this systematic fragmentation of DNA is the cleavage of poly (ADP-ribose) polymerase (PARP) into its inactive cleaved form [36], a process that ultimately deters the DNA damage repair system [37]. Our protein blot results show an upregulation in the expression of c-PARP in MDA-MB-231 and MCF-7 cells with increased ScBEE concentrations, which explains the effect of the extract on DNA fragmentation. Several studies have demonstrated the potent activity of secondary plant metabolites in the prevention and treatment of cancer cells [38]. In a study conducted by Nanni et al., an extract preparation rich in phenols and flavonoids that was also detected in our extract, was found to induce DNA damage in human melanoma A375 cells via the mitochondrial apoptotic pathway, similarly to our findings in BC cells [39]. To further investigate the apoptotic pathway initiated by ScBEE in each of the BC cell lines, the expression of other protein mediators was analyzed through Western blots. One of the major extrinsic apoptotic pathway executers [40], caspase 8 in its active cleaved form, was shown to have a constant expression with increased treatment concentration in both MDA-MB-231 and MCF-7 cells. Other pro- and antiapoptotic proteins that dictate cellular fate through the inhibition or progression of the intrinsic apoptotic pathway are members of the Bcl-2 family [41], the expression of which was also measured in order to check their involvement in the programmed death triggered by ScBEE. Our blots showed a significant increase in the ratio of the expression of proapoptotic Bax protein relative to that of anti-apoptotic Bcl-2 in both ScBEE-treated BC cell lines. These results confirm that the antiproliferative effect of ScBEE is particularly mediated through the intrinsic apoptotic pathway.

In their previous study that aimed to determine the phenolic content and antioxidant promise of one *Sternbergia* species, Aydin et al. demonstrated that ethanolic extract of *Sternbergia lutea* bulbs exhibited a significant total antioxidant effect with prominent free radical scavenging activity [42]. Our results showed a similar effect of ScBEE on BC cells that exhibited a significant inhibition of ROS levels with increased treatment concentrations. Despite the contentious role of ROS in modulating apoptosis, several natural compounds with a prominent antioxidant potential were shown to acquire a cell-line-dependent proapoptotic effect [43]. Therefore, in order to determine ScBEE components that are responsible for this antioxidant cell-death- inducing effect, chemical characterization of the extract via LC-MS was performed, and the identity of the bioactive compounds was revealed.

Abundant molecules that were found in ScBEE belong to the families of benzofurans, alkaloids, linoleic acid derivatives, quinoline derivatives, phenols, and flavonoids. This variety of chemical constituents suggests a broad activity spectrum resulting from a specific mode of action on each of the identified compounds. Among the antioxidant compounds detected in ScBEE, D-(−)-quinic acid and the flavonoid derivative luteolin were previously shown to exert proapoptotic effect on oral cancer cells (squamous cell carcinoma SCC-4) [44,45] and human colorectal cancer cells (HT-29) [46], whereas another flavonoid-rich extract was found to promote apoptosis via the p53-dependent mitochondrial pathway in A549 cells [47].

The potential of ScBEE to induce apoptosis through the modulation of p53 was not investigated in this study; however, several flavonols detected in our extract were previously shown to upregulate the expression of many tumor suppressors involved in apoptotic cell death and chemoprevention [48]. In a study conducted by Lan et al., ScBEE flavonol derivative rhamnetin was identified to exhibit apoptotic activity, which is tightly associated with the overexpression of p53 and its downstream target, miR-34a, in MCF-7 cells, in addition to the downregulation of breast cancer promoter Notch1 protein [49].

The flavonol quercetin is a potent ROS scavenger detected in our extract and that was previously shown to cause DNA damage [50] and promote apoptosis via the activation of the estrogen receptor α-dependent p38 signal transduction pathway [51], a mechanism that could explain the proapoptotic effect of ScBEE in ER-expressing cells (MCF-7) but not in ER-negative cells (MDA-MB-231) [3], where another ER-independent apoptotic pathway was activated. Several compounds detected by LC-MS/MS in ScBEE were shown to inhibit cancer cell proliferation through the activation of a wide variety of ER-independent intrinsic pathways. These compounds involve the linoleic acid derivatives jasmonic and corchorifatty acids previously shown to stimulate the mitochondrial apoptotic pathway [52,53] that is tightly associated with the expression ratio of Bax/Bcl2 [54], an index that was shown to be upregulated in ScBEE-treated breast cancer cells. The two cell lines demonstrated a similar response to ScBEE treatment, although they are genetically different. As mentioned previously, this could be explained by the presence of various phytochemical compounds that were reported to act differently on breast cancer cell lines while activating the mitochondrial-dependent apoptotic pathway in MDA-MB-231 and MCF-7. Several studies in the literature have reported that ER(+) and ER(−) cells might undergo apoptosis via the same mechanism of action, regardless of the difference in their genetic profile [55,56].

The flavonoid derivative luteolin, which was previously described for its anticancer activity via the activation of the apoptotic pathway, was also evaluated for its antioxidant activity. A recent study by Kang et al. determined the mitochondrial-dependent apoptotic effect of luteolin on HT-29 cancer cells through its antioxidant properties. The authors demonstrated the activation of the intrinsic apoptotic mechanism via the downregulation of Bcl2 and the upregulation of Bax, inducing cytochrome c release from the mitochondria. This luteolin-induced apoptosis was demonstrated to be accompanied by the activation of several antioxidant enzymes [46]. This might define the activation of the intrinsic apoptotic pathway mediated by the antioxidant properties of flavonoid compounds and its derivatives as detected by LC-MS analysis of the ScBEE extract. This could be further explained by another study conducted on flavonoids in vitro in which an ROS-independent activation of the mitochondrial pathway was demonstrated in HL-60 cells [57]. Moreover, one of the most abundant compounds identified in ScBEE was the Amaryllidaceae alkaloid lycorine, a constituent with an important tumoricidal effect against breast cancer cells, as previously reported in studies by Wang et al. [58] and Ying et al. [59]. The cytotoxicity of lycorine was revealed in both MDA-MB-231 and MCF-7 cells, an effect that was confirmed in vitro and in vivo, accompanied by the downregulation of the expression of antiapoptotic proteins, such as Bcl-2 family proteins, and the upregulation of proapoptotic c-PARP. The apoptotic potential on BC cells was also shown to be exerted by the terpenoid trans-anethole [60], the phenolic compound ferulic acid [61] and most of the benzofuran derivatives [62] detected in ScBEE.

## 4. Materials and Methods

### 4.1. Plant Material

*Sternbergia clusiana* (Ker Gawl.) plants were collected from Falougha, Lebanon (33.825008° N, 35.751962° E, 1497 m above sea level) during November 2018 and identified by the botanist and expert on Lebanese flora, Dr. Nisrine Machaka-Houri (Saint Joseph University, Lebanon). A voucher specimen (ID: Machaka 81) was deposited at the Post Herbarium of the American University of Beirut, Lebanon.

### 4.2. Sternbergia Bulb Ethanolic Extract (ScBEE) Preparation

After collecting S. *clusiana*, the bulbs were washed with distilled water, dried and ground. A mass of 36.47 g of ground bulbs was mixed in 350 mL of ethanol (70%) and thermo-shaken at 200 rpm for one week at room temperature (25 °C). Ethanol was evaporated from a volume of 50 mL of the mixture by applying mechanical rotation under vacuum (roto-evaporation). The extract was redissolved under sonication in 2.5 mL DMSO and 25 mL of cell culture medium (10% DMSO). The prepared solution was filtered using a sterile cheese cloth, and the volume was brought to 90 mL. The solution was later centrifuged at 24,446× *g* and filtered with a syringe filter (0.45 µm) to a final volume of 87 mL. The pure extract (232.7 µg/mL) was labeled ScBEE, aliquoted and stored at −80 °C for later use. Each aliquot (10% DMSO) was diluted 10 times in DMEM prior to treatment, maintaining a final concentration of DMSO less than 0.1%. BC cells were exposed to 0.0015, 0.003, 0.005, 0.015, 0.025 and 0.035% DMSO upon treatment with 3.49, 6.98, 11.64, 23.27, 58.18 and 81.45 μg/mL (0.15, 0.3, 0.5, 1.5, 2.5 and 3.5% *v*/*v*) ScBEE, respectively.

### 4.3. Breast Cancer Cell Culture

Two BC cell lines, MDA-MB-231 (ER^-^, PR^-^, HER2^-^) and MCF7 (ER^+^), were obtained from ATCC for culture and further experimentation. Both MDA-MB-231 and MCF7 were established from a pleural effusion of two distinct Caucasian females aged 51 and 69 years, respectively, suffering from metastatic mammary adenocarcinoma. The cells were cultured in a humidified incubator (37 °C, 5% CO_2_) using Dulbecco’s modified eagle medium (DMEM) supplemented with 10% fetal bovine serum (FBS) and antibiotics (100 U/mL penicillin and 100 µg/mL streptomycin) [63]. The cells were split every 3 days at 70–80% confluency using phosphate-buffered saline (PBS) for washing and trypsin-EDTA for detachment. Cell morphology and viability were regularly checked prior to each experiment using ZOE fluorescent cell imager and trypan exclusion method, respectively.

### 4.4. Culture of Mesenchymal Stem Cells (MSCs) Isolated from Rat Bone Marrow

Following the Lebanese American University’s (LAU) Animal Care and Use Committee (ACUC.- SAS.CD4.1) guidelines and the Guide for the Care and Use of Laboratory Animals (Committee for the Update of the Guide for the Care and Use of Laboratory Animals, 2010, LAU), MSC cells were isolated from rat bone marrow (BM). The animal facility at the Lebanese American University provided us with a 12-week-old rat that was sacrificed under CO_2_ asphyxiation. As previously detailed by Haykal et al., femoral and tibial bones were aseptically isolated and washed [64]. After removing the bone epiphyses with sterilized scissors, the BM was flushed out, and the cells were collected and incubated in a vented flask at 37 °C and a 5% CO_2_ incubator. After 5 days of daily medium change, MSCs were identified by their spindle-shaped morphology using a ZOE fluorescent cell imager. All procedures were carried out in accordance with the ARRIVE guidelines (https://arriveguidelines.org) accessed on 14 August 2022.

### 4.5. Cytotoxicity Assay

MDA-MB-231 and MCF7 cells were seeded in 96-well plates at a confluency of 1 × 10^5^ cells/mL, then treated with increasing concentrations of ScBEE (3.49, 6.98, 11.64, 23.27, 58.18 and 81.45 μg/mL) for 24 and 48 h. Similarly, MSCs were seeded in a 96-well plate and treated with high doses of ScBEE (232.7, 698.1 and 1163 μg/mL) for 24 h. In the presence of PMS, MTS (Promega) is reduced into a brown formazan product through NADPH-dependent dehydrogenase enzymes found in metabolically active cells. Cell viability was measured using a Varioskan™ LUX multimode microplate reader (Thermo Fisher Scientific, Bremen, Germany) at a wavelength of 492 nm [65]. Percent proliferation was calculated and IC_50_ values were determined using GraphPad Prism 8.

### 4.6. Cell Cycle Analysis

MDA-MB-231 and MCF-7 cells (2 × 10^5^ cells/mL) were seeded in 6-well plates and treated with increasing concentrations of ScBEE (3.49, 11.64 and 58.18 μg/mL) for 24 h. Cells were collected and fixed as previously described by Idris et al. [66]. The following day, cells were centrifuged (736.09× *g*, 10 min, 4 °C), counted using trypan blue and stained with 50 μg/mL PI and 0.45 μg/mL RNase [67]. The DNA content was analyzed and classified using a Guava easyCyte™ flow cytometer depending on the degree of PI binding as follows: sub-G0/G1 phase cells (pre-G or dead cells) have <2 n, G0/G1 phase cells have 2 n, S phase cells have between 2 n and 4 n and G2/M phase cells have 4 n.

### 4.7. Cell Death ELISA

Both BC cell lines were seeded at a confluency of 2 × 10^5^ cells/mL overnight in 6-well plates, then treated with increasing concentrations of ScBEE (3.49, 11.64 and 58.18 μg/mL), and topotecan (20 µM) or cisplatin (30 µM) were used as positive controls for 24 h. The following day, cells were washed, detached and lysed according to the manufacturer’s instructions. The DNA-rich supernatant was plated into a histone-coated microplate (prepared and left overnight at 4 °C) [68]. DNA fragmentation was measured using a Varioskan™ LUX multimode microplate reader, and the enrichment factor of fragmented DNA was calculated as the ratio of absorbance in the treated samples to that of the untreated control.

### 4.8. Protein Extraction and Quantification

MDA-MB-231 and MCF-7 cells were plated in Petri dishes (2 × 10^5^ cells/mL and 3 × 10^5^ cells/mL, respectively) overnight, then treated with increasing concentrations of ScBEE (3.49, 11.64 and 58.18 μg/mL) for 24 h. Cells were lysed on ice using lysis buffer from a Qproteome mammalian protein prep kit (Qiagen, Hilden, Germany), then quantified using a detergent-compatible (DC) protein assay (Bio-Rad, Hercules, CA, USA).

### 4.9. Western Blot

Extracted proteins were then separated by SDS-PAGE (10%) and transferred to PVDF membranes. The membranes were blocked as previously described by Khalife et al. and incubated with primary antibodies overnight: anti-β-actin (Santa Cruz Biotechnology, Dallas, TX, USA) was used as a loading control, in addition to anti-cleaved poly (ADP-ribose) polymerase (PARP) (Abcam, Cambridge, UK), anti-Caspase-8 (Elabscience, Houston, TX, USA) and anti-Bcl2 (Elabscience, Houston, TX, USA) [69]. Membranes were washed to remove unspecific binding and incubated for another hour with the specific secondary antibody (Bio-Rad, Irvine, CA, USA) [70]. Images were developed and quantified in order to calculate the relative protein expression using ImageJ.

### 4.10. Reactive Oxygen Species (ROS) Detection

MDA-MB-231 and MCF-7 cells were plated at a density of 1 × 10^5^ cells/mL in 96-well plates. A DCFDA cellular ROS detection assay kit (Abcam, Cambridge, UK) was used in to quantity the level of ROS upon treatment with ScBEE. Briefly, the cells were preincubated with 20,70-dichlorodihydrofluorescein diacetate (H2DCFDA) reagent; then, increasing concentrations of the extract were added [8]. TBHP (30 µM), a potent ROS inducer, and NAC (1 mM), a potent ROS inhibitor, were used as positive and negative controls, respectively. As a result of ROS production, the amount of H2DCFDA oxidized into DCF was measured using a Varioskan™ LUX multimode microplate reader (Thermo Fisher Scientific, Bremen, Germany) [70].

### 4.11. Liquid Chromatograph-Tandem Mass Spectrometry (LC-MS/MS) Analysis

The chemical composition of ScBEE was analyzed as previously described [8]. Briefly, the sample (2.5 µg) was injected into a C18 Gravity-SB Nucleodur 300 Å, 1.8 µm, 2 mm × 100 mm (Macherey-Nagel, Düren, Germany) column using a Dionex Ultimate 3000 analytical RSLC system (Dionex, Germering, Germany) coupled with a heated electrospray HESI source (Thermo Fisher Scientific, Bremen, Germany). The separation was performed with flow rate of 300 µL/min by applying a gradient of solvent B from 3 to 50% within 35 min, followed by column washing and re-equilibration steps. Solvent A was composed of water with 0.1% formic acid, whereas solvent B consisted of acetonitrile with 0.1% formic acid. Eluting compounds were analyzed on a QExactive HF-HT-Orbitrap-FT-MS benchtop instrument (Thermo Fisher Scientific, Bremen, Germany) either in positive or negative polarity. An MS1 scan was performed with 60,000 resolution, AGC (automatic gain control) of 3e6 and a maximum injection time of 200 ms. An MS2 scan was performed in Top10 mode with a 2 *m*/*z* isolation window, AGC of 5e5, 15,000 resolution, a maximum injection time of 50 ms and an average of 2 µ scans. High-energy collisional dissociation (HCD) was used as fragmentation method with normalized collision energy of 28%. The raw data were processed for both positive and negative polarities using Compound Discoverer^TM^ 3.2 software (Thermo Fisher Scientific, Bremen, Germany). Corresponding blank samples were used for background signal subtraction and noise removal in the pre-processing step. A custom-designed workflow was established for spectral alignment, compound detection, grouping and metabolite identification using mzVault, mzCloud and ChemSpider databases.

### 4.12. Statistical Analysis

All experiments were carried out in triplicate, and each experiment was performed at least three times (n = 3). Statistical analyses were performed using GraphPad Prism 8, and data are reported as mean values ± SD. To calculate *p*-values, a *t*-test or two-way ANOVA was used, depending on the experiment. Significant differences are represented; * indicates a *p*-value of 0.01 < *p* < 0.05, ** indicates a *p*-value of 0.001 < *p* < 0.01 and *** indicates a *p*-value of *p* < 0.001.

## 5. Conclusions

The current study reveals a promising proapoptotic and antioxidant effect of *Sternbergia clusiana* bulb ethanolic extract on triple-negative and estrogen-dependent breast cancer cell lines in vitro. The data presented herein validate the chemopreventive properties of this extract via the activation of the mitochondrial pathway, as revealed by the upregulation of the Bax/Bcl2 ratio, along with an increase in major apoptotic hallmarks such as cellular and DNA fragmentation. Because ScBEE was identified for its chemical composition, the antioxidant and anticancer activities of this extract may have contributed to its phytochemical constituents, representing a potential target for future work.

## Figures and Tables

**Table 1:** IC_50_ of MDA-MB-231 and MCF-7 cells after 24 and 48 h incubation with ScBEE.

	IC_50_ Value	
Breast Cancer Cell Line	24 h	48 h
MDA-MB-231	0.926% *v*/*v* (≈21.55 µg/mL)	0.289% *v*/*v* (≈6.73 µg/mL)
MCF-7	0.992% *v*/*v* (≈23.08 µg/mL)	0.160% *v*/*v* (≈3.72 µg/mL)

**Table 2:** Chemical characterization of ScBEE by LC-MS/MS.

Name	Formula	RT (min)	Area (max)	Negative Run	Positive Run
Bis(4-ethylbenzylidene) sorbitol	C_24_H_30_O_6_	37.878	3,174,434,618		3,174,434,618
4-oxo-4,5,6,7-tetrahydrobenzo[b]furan-3-carboxylic acid	C_9_H_8_O_4_	11.438	2,230,875,723	2,230,875,723	
Lycorine	C_16_H_17_NO_4_	2.837	2,150,797,725		2,150,797,725
3-oxoindane-1-carboxylic acid	C_10_H_8_O_3_	31.823	1,490,616,529		1,490,616,529
L-Phenylalanine	C_9_H_11_NO_2_	2.793	1,081,816,069		1,081,816,069
(9*Z*,11*E*,15*Z*)-13-hydroxyoctadeca-9,11,15-trienoic acid	C_18_H_30_O_3_	38.526	926,272,408.7		926,272,408.7
(10*E*,12*E*)-9-hydroperoxyoctadeca-10,12-dienoic acid	C_18_H_32_O_4_	38.535	890,320,782.9	890,320,782.9	
2-Amino-1,3,4-octadecanetriol	C_18_H_39_NO_3_	31.808	610,188,836.2		610,188,836.2
13,14-dihydro Prostaglandin F1α	C_20_H_38_O_5_	35.606	511,502,894.1	511,502,894.1	
9S,13R-12-Oxophytodienoic acid	C_18_H_28_O_3_	39.803	461,820,295.2	68,310,411.97	461,820,295.2
Cetrimonium	C_19_H_41_N	39.798	420,064,536.9		420,064,536.9
3-hydroxy-4-(3-hydroxyphenyl)-1,2-dihydroquinolin-2-one	C_15_H_11_NO_3_	9.26	308,498,766.5		308,498,766.5
4-Hydroxybenzoic acid	C_7_H_6_O_3_	36.532	297,387,086.7		297,387,086.7
D-(+)-Tryptophan	C_11_H_12_N_2_O_2_	5.543	253,102,825.1	155,006,313.2	253,102,825.1
D-(−)-Quinic acid	C_7_H_12_O_6_	3.174	247,235,220.9	247,235,220.9	
4-(tert-butyl)phenyl 3,5-dimethylisoxazole-4-carboxylate	C_16_H_19_NO_3_	3.226	240,995,401.5		240,995,401.5
Sedanolide	C_12_H_18_O_2_	38.527	183,054,018.2		183,054,018.2
Corchorifatty acid F	C_18_H_32_O_5_	31.371	178,994,397.1	178,994,397.1	
2,2,6,6-Tetramethyl-1-piperidinol (TEMPO)	C_9_H_19_NO	29.349	164,033,305.7		164,033,305.7
α-Hydroxymidazolam	C_18_H_13_ClFN_3_O	10.109	104,041,359.5		104,041,359.5
Ferulic acid	C_10_H_10_O_4_	11.449	101,224,790.3		101,224,790.3
Rhamnetin	C_16_H_12_O_7_	18.912	96,025,818.23		96,025,818.23
4-Indolecarbaldehyde	C_9_H_7_NO	16.502	79,507,735.41		79,507,735.41
3-Methoxy-5,7,3’,4’-tetrahydroxy-flavone	C_16_H_12_O_7_	27.133	79,447,955.04	79,447,955.04	78,747,838.78
Isoleucine	C_6_H_13_NO_2_	2.327	71,402,978.66		71,402,978.66
L-Pyroglutamic acid	C_5_H_7_NO_3_	2.204	61,948,424.66		61,948,424.66
L-Phenylalanine	C_9_H_11_NO_2_	2.135	61,341,499.61		61,341,499.61
Corchorifatty acid F	C_18_H_32_O_5_	26.367	60,847,215.25	60,847,215.25	
ethyl 9H-beta-carboline-3-carboxylate	C_14_H_12_N_2_O_2_	19.841	56,707,514.56		56,707,514.56
Phomolide G	C_12_H_20_O_5_	19.341	54,163,834.9	54,163,834.9	
Cinchophen	C_16_H_11_NO_2_	10.536	40,663,903.34		40,663,903.34
9S,13R-12-Oxophytodienoic acid	C_18_H_28_O_3_	31.311	39,636,902.48		39,636,902.48
4-Acetyl-3-hydroxy-5-methylphenyl β-D-glucopyranoside	C_15_H_20_O_8_	8.52	39,273,094.27	39,273,094.27	
trans-Cinnamaldehyde	C_9_H_8_O	11.449	38,739,571.25		38,739,571.25
Quercetin	C_15_H_10_O_7_	16.992	35,465,633.57		35,465,633.57
3,4-Dihydroxybenzaldehyde	C_7_H_6_O_3_	8.401	33,254,282.78		33,254,282.78
Jasmonic acid	C_12_H_18_O_3_	24.837	31,224,896.2	31,224,896.2	
Corchorifatty acid F	C_18_H_32_O_5_	36.331	29,858,068.04	29,858,068.04	
Pyridoxal	C_8_H_9_NO_3_	15.696	29,711,710.82		29,711,710.82
4-Indolecarbaldehyde	C_9_H_7_NO	5.467	29,689,856.04		29,689,856.04
2-benzyl-6-hydroxy-2-azabicyclo [2.2.2]octan-3-one	C_14_H_17_NO_2_	2.876	25,424,100.69		25,424,100.69
Sedanolide	C_12_H_18_O_2_	16.589	25,382,213.3		25,382,213.3
Azelaic acid	C_9_H_16_O_4_	18.216	23,684,670.17	23,684,670.17	
L-Norleucine	C_6_H_13_NO_2_	2.884	17,292,410.45		17,292,410.45
Sedanolide	C_12_H_18_O_2_	27.958	15,283,130.43		15,283,130.43
trans-Anethole	C_10_H_12_O	34.624	15,280,838.86		15,280,838.86
Tetradecanedioic acid	C_14_H_26_O_4_	29.245	14,533,711.98	14,533,711.98	
Adenosine	C_10_H_13_N_5_O_4_	2.633	14,190,960.27		14,190,960.27
(±)-Abscisic acid	C_15_H_20_O_4_	21.622	12,847,644.97	12,847,644.97	
Methyl cinnamate	C_10_H_10_O_2_	13.745	12,569,420.87		12,569,420.87
Fumaritine N-oxide	C_20_H_21_NO_6_	10.14	12,365,381.38		12,365,381.38
Melicopidine	C_17_H_15_NO_5_	18.576	12,285,467.72		12,285,467.72
Luteolin-3’,7-Diglucoside	C_27_H_30_O_16_	12.912	10,648,923.76	8,681,866.882	10,648,923.76

## Data Availability

All data generated and analyzed in this study are mentioned in this manuscript.

## Supplementary Materials

The following supporting information can be downloaded at: https://www.mdpi.com/article/10.3390/plants12030529/s1, Figure S1: Full length blots of MDA MB 231 reported in the manuscript in Figure 3C; Figure S2: Full length blots of MCF 7 reported in the manuscript in Figure 3D; Figure S3: Full length blots of MDA MB 231 reported in the manuscript in Figure 4A; Figure S4: Full length blots of MDA MB 231 reported in the manuscript in Figure 4A; Figure S5: Full length blots of MCF 7 reported in the manuscript in Figure 4B; Figure S6: Full length blots of MCF 7 reported in the manuscript in Figure 4B; Figure S7: Full length blots of MDA MB 231 reported in the manuscript in Figure 5; Figure S8: Full length blots of MCF 7 reported in the manuscript in Figure 5. In Trial 2, we had to cover the top part of the blot and increase the exposure to be able to see the cleaved form of caspase 8 due to overexpression of the upper bands. Table S1: Targeted identification of alklaoid derivatives in ScBEE using LCMS/MS.
