# Supplementary material for: The Antioxidant and Proapoptotic Effects of Sternbergia clusiana Bulb Ethanolic Extract on Triple-Negative and Estrogen-Dependent Breast Cancer Cells In Vitro
Source: Plants (Basel). 2023 Jan 24;12(3):529. doi: 10.3390/plants12030529 (PMC9920827; doi:10.3390/plants12030529)
Supplement: Supplementary file 1 [file plants-12-00529-s001.zip › Supplementary figures of full length blots.pdf]

The Antioxidant and Proapoptotic Effects of *Sternbergia clusiana* Bulb Ethanolic Extract on Triple-Negative and Estrogen-Dependent Breast Cancer Cells In Vitro

Mona El Samarji [1,#], Maria Younes [1,#], Marianne El Khoury [1], Tony Haykal [1], Nazira Elias [1],
Natalia Gasilova [2], Laure Menin [2], Ahmad Houri [3], Nisrine Machaka-Houri [4], Sandra Rizk [1,*]

Supplementary information

Full length blots reported in Figures 3,4 and 5

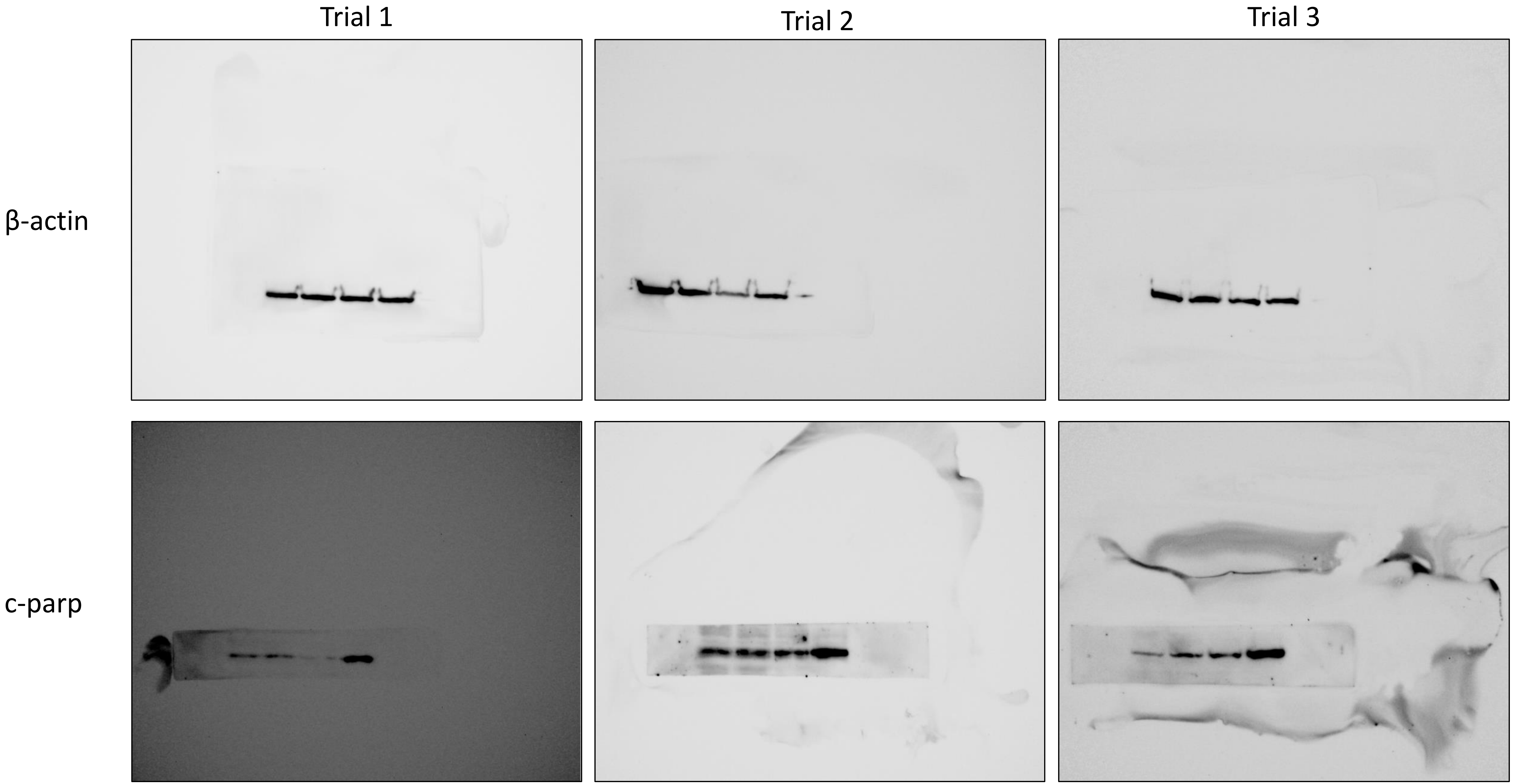

Supplementary Figure S1: Full length blots of MDA-MB-231 reported in the manuscript in Fig.3C

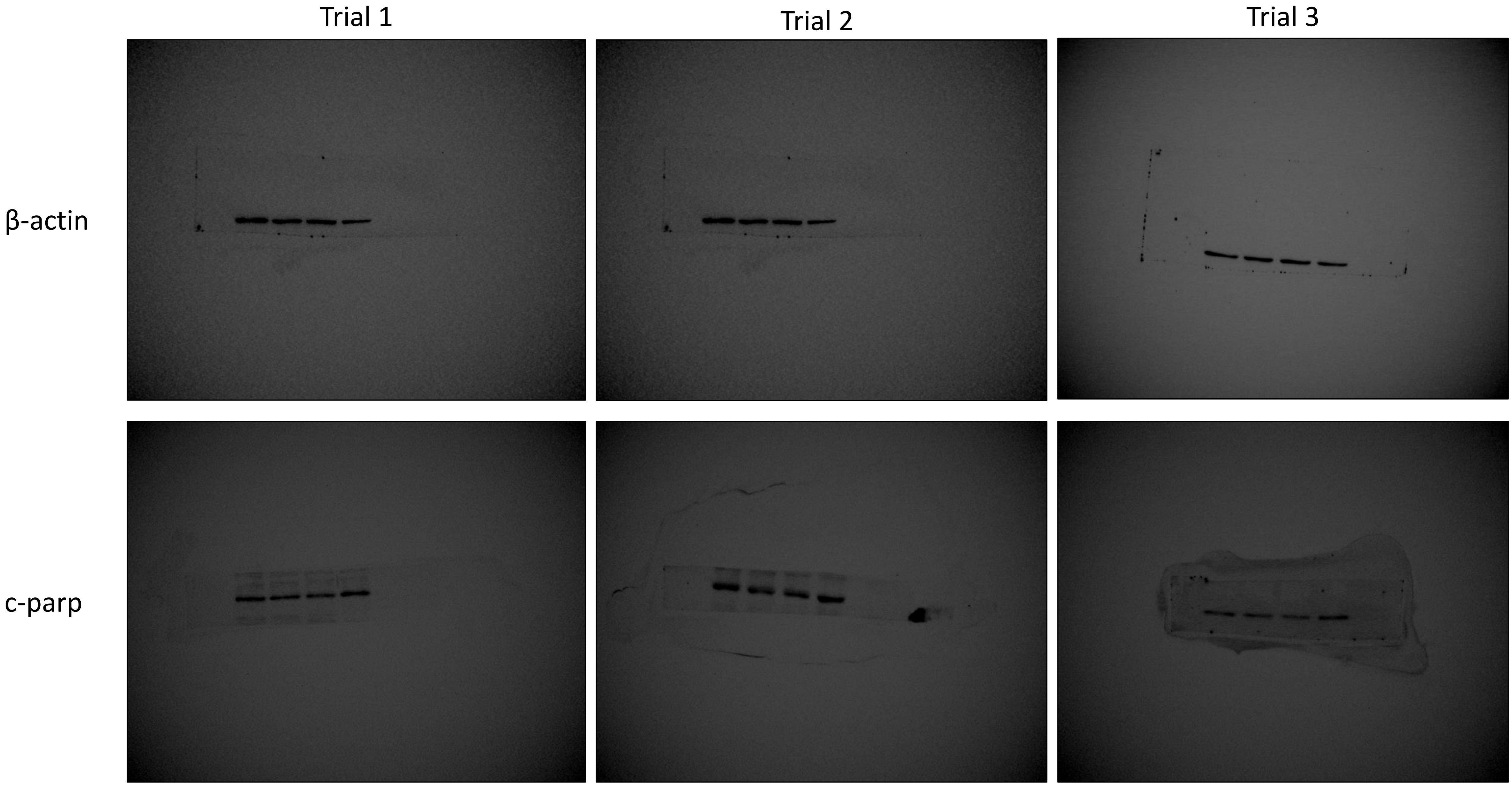

Supplementary Figure S2: Full length blots of MCF-7 reported in the manuscript in Fig.3D

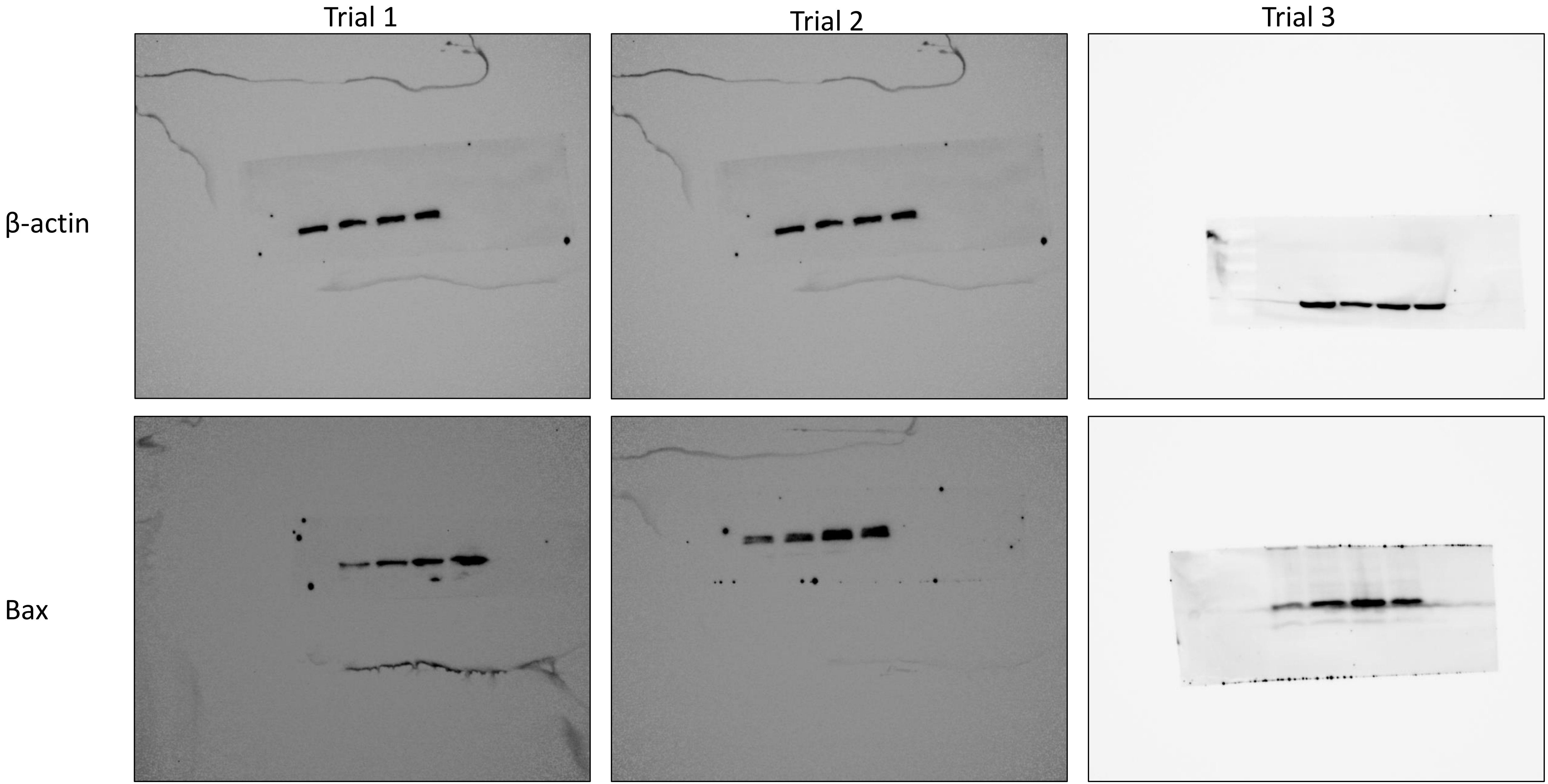

Supplementary Figure S3: Full length blots of MDA-MB-231 reported in the manuscript in Fig.4A

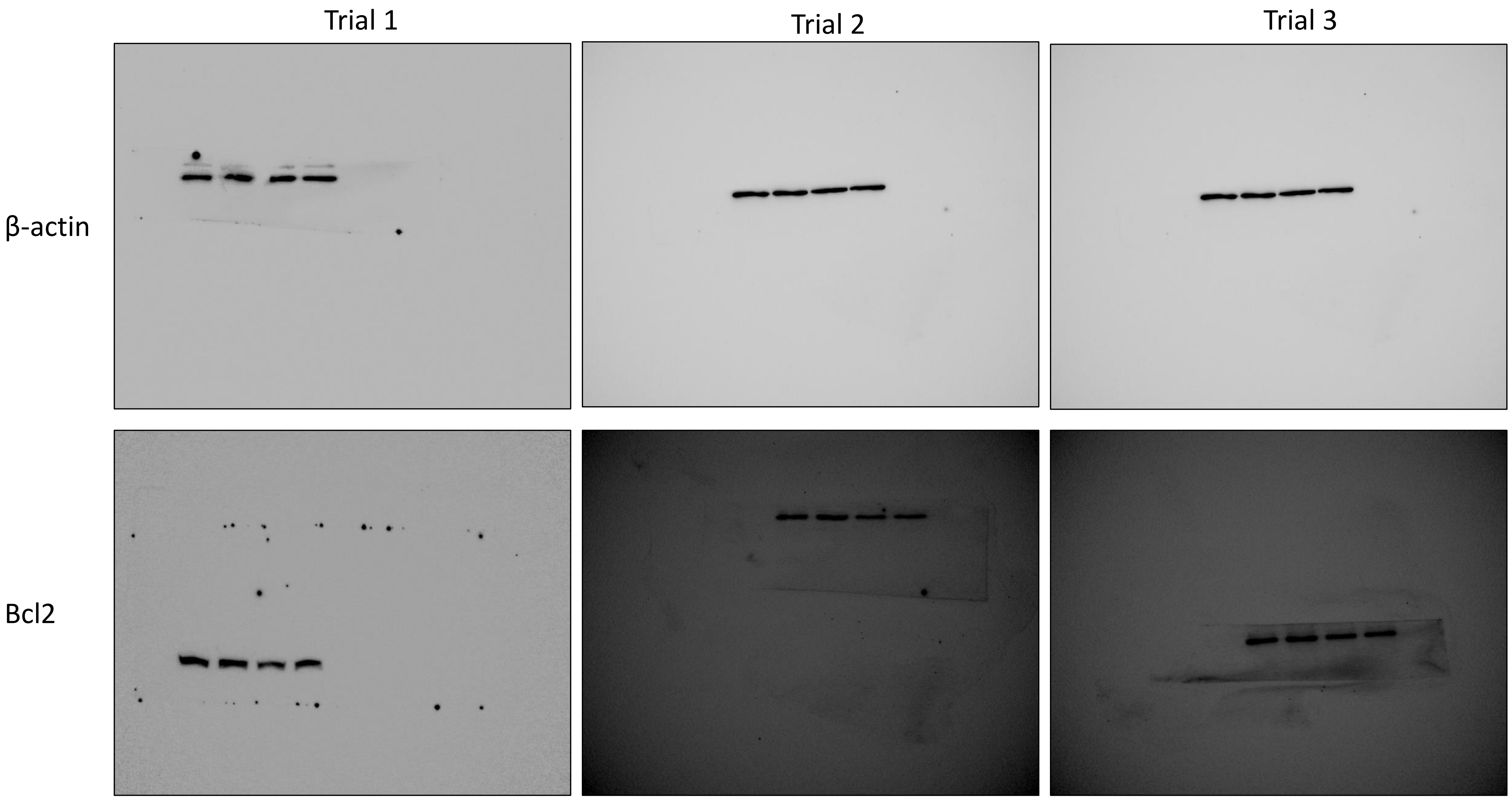

Supplementary Figure S4: Full length blots of MDA-MB-231 reported in the manuscript in Fig.4A

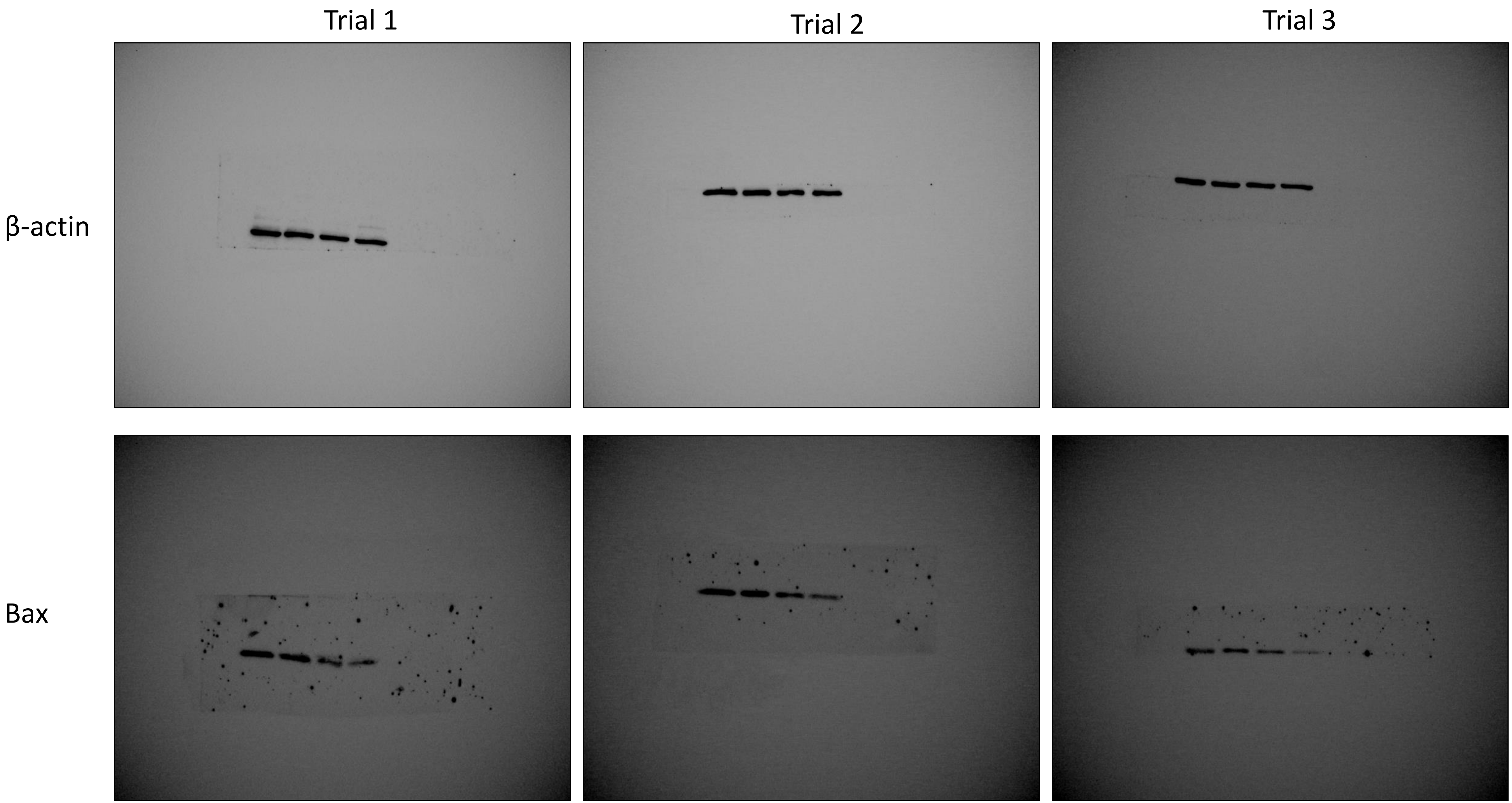

Supplementary Figure S5: Full length blots of MCF-7 reported in the manuscript in Fig.4B

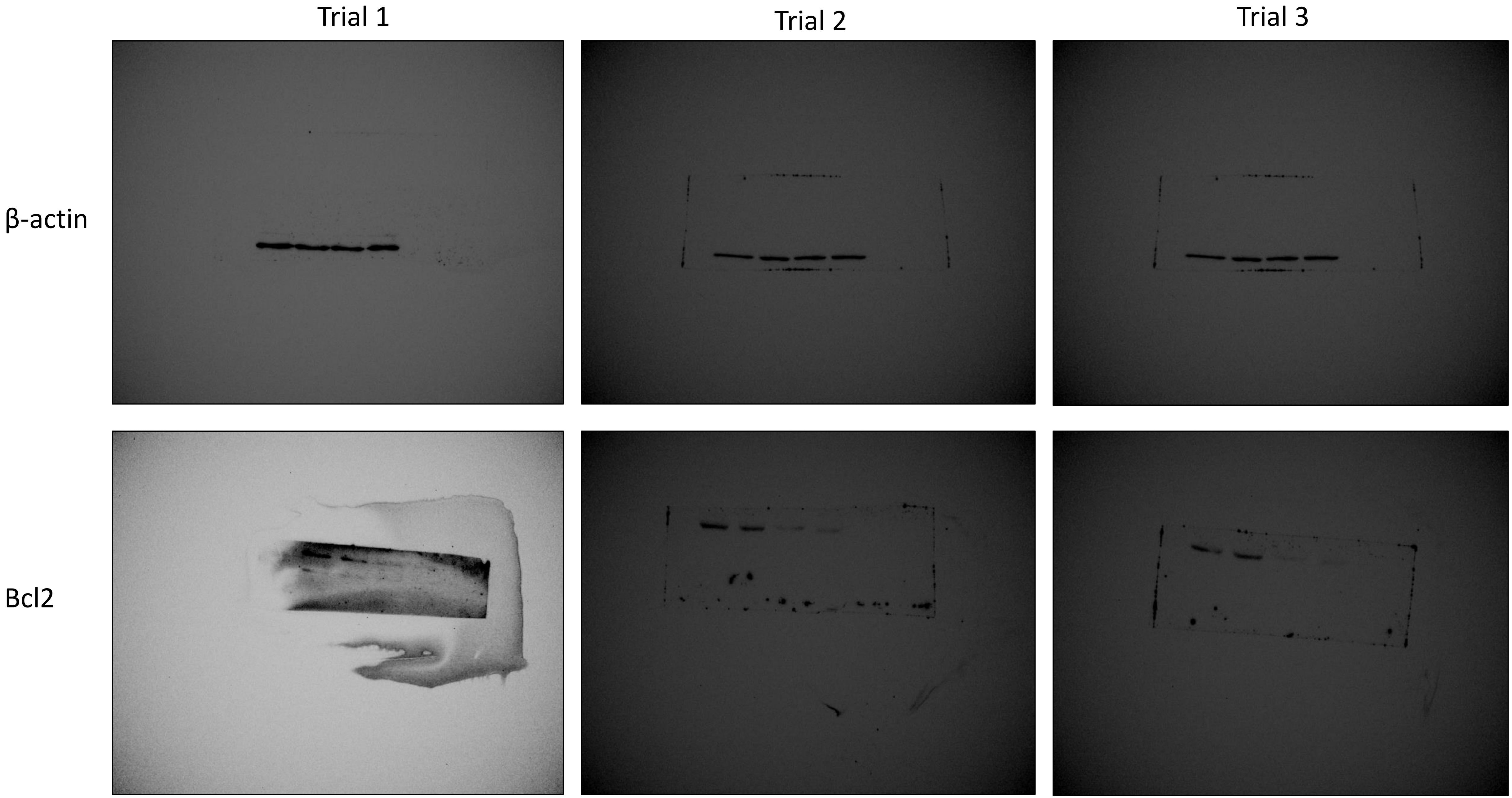

Supplementary Figure S6: Full length blots of MCF-7 reported in the manuscript in Fig.4B

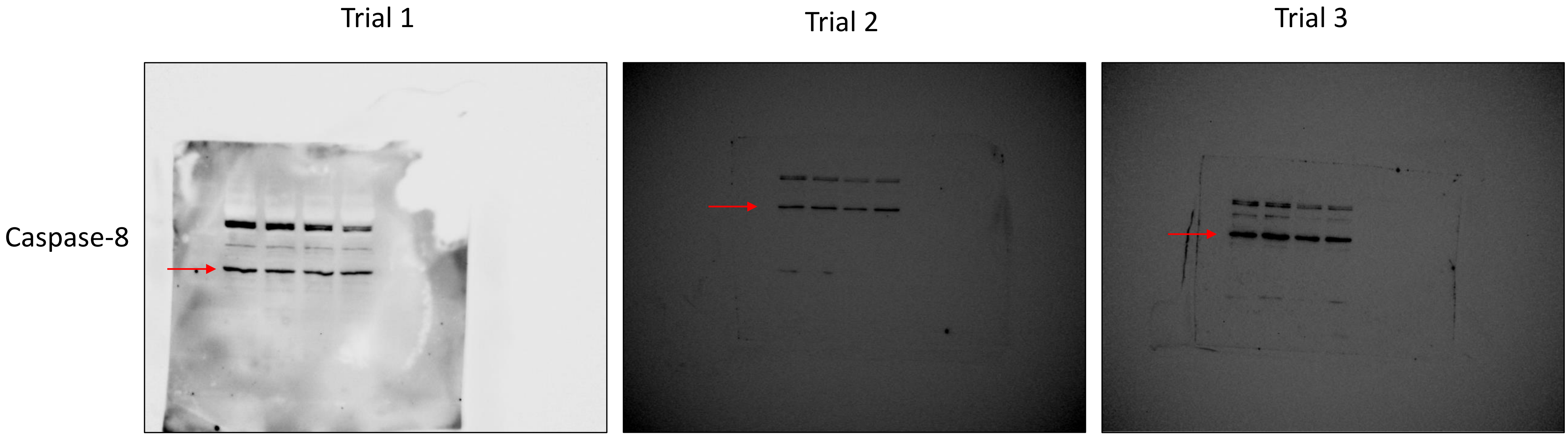

Supplementary Figure S7: Full length blots of MDA-MB-231 reported in the manuscript in Fig.5

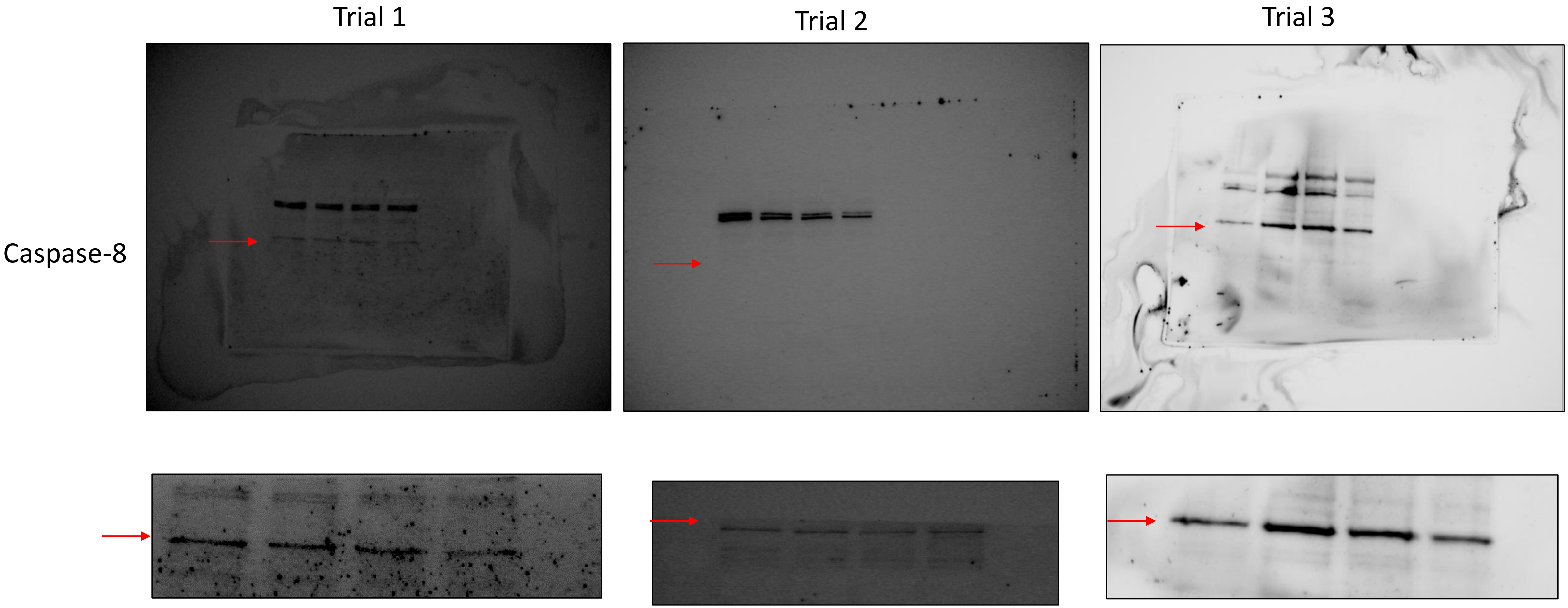

Supplementary Figure S8: Full length blots of MCF-7 reported in the manuscript in Fig.5. In Trial 2, we had to cover the top part of the blot and increase the exposure to be able to see the cleaved form of caspase-8 due to overexpression of the upper bands.
